# The rib cage stabilizes the human thoracic spine: An *in vitro* study using stepwise reduction of rib cage structures

**DOI:** 10.1371/journal.pone.0178733

**Published:** 2017-06-01

**Authors:** Christian Liebsch, Nicolas Graf, Konrad Appelt, Hans-Joachim Wilke

**Affiliations:** Institute of Orthopaedic Research and Biomechanics, Trauma Research Centre Ulm, Ulm University, Ulm, Germany; University of Zaragoza, SPAIN

## Abstract

The stabilizing effect of the rib cage on the human thoracic spine is still not sufficiently analyzed. For a better understanding of this effect as well as the calibration and validation of numerical models of the thoracic spine, experimental biomechanics data is required. This study aimed to determine (1) the stabilizing effect of the single rib cage structures on the human thoracic spine as well as the effect of the rib cage on (2) the flexibility of the single motion segments and (3) coupled motion behavior of the thoracic spine. Six human thoracic spine specimens including the entire rib cage were loaded quasi-statically with pure moments of ± 2 Nm in flexion/extension (FE), lateral bending (LB), and axial rotation (AR) using a custom-built spine tester. Motion analysis was performed using an optical motion tracking system during load application to determine range of motion (ROM) and neutral zone (NZ). Specimens were tested (1) in intact condition, (2) after removal of the intercostal muscles, (3) after median sternotomy, after removal of (4) the anterior rib cage up to the rib stumps, (5) the right sixth to eighth rib head, and (6) all rib heads. Significant (p < 0.05) increases of the ROM were found after dissecting the intercostal muscles (LB: + 22.4%, AR: + 22.6%), the anterior part of the rib cage (FE: + 21.1%, LB: + 10.9%, AR: + 72.5%), and all rib heads (AR: + 5.8%) relative to its previous condition. Compared to the intact condition, ROM and NZ increased significantly after removing the anterior part of the rib cage (FE: + 52.2%, + 45.6%; LB: + 42.0%, + 54.0%; AR: + 94.4%, + 187.8%). Median sternotomy (FE: + 11.9%, AR: + 21.9%) and partial costovertebral release (AR: + 11.7%) significantly increased the ROM relative to its previous condition. Removing the entire rib cage increased both monosegmental and coupled motion ROM, but did not alter the qualitative motion behavior. The rib cage has a strong effect on thoracic spine rigidity, especially in axial rotation by a factor of more than two, and should therefore be considered in clinical scenarios, in vitro, and in silico.

## Introduction

The biomechanical properties of the human thoracic spine are still largely unknown, since spinal research has mainly focused on the lumbar and cervical spine in the past. Especially the influence of the anterior rib cage structures on the stiffness and motion behavior of the thoracic spine is poorly investigated. Detailed knowledge of the thoracic spine biomechanics including the rib cage is required for improving surgical and conservative treatments of the thoracic spine, for instance in the case of scoliosis or hyperkyphosis. Numerical models of the thoracic spine and rib cage therefore provide a useful tool. In order to validate these models, kinematics data and stiffness properties of the thoracic spine including the rib cage are essential.

Since the validation of numerical models is based on the design of the experimental test setup, the results of former in silico studies varied considerably [1, 2]. A clear definition of initial and boundary conditions is therefore of high importance in order to obtain long lasting and impactful data. An established method for characterization of spinal stiffness properties and data collection for the validation of numerical models is provided by the concept of stepwise reduction of single anatomical structures. This concept was mainly applied to single motion segments of the cervical and lumbar spine in the past [3–6]. The first in vitro experiments quantifying the effect of the rib cage structures on thoracic spine stability were performed on canine specimens, in which the effects of the posterior elements, costovertebral joints, and intervertebral discs were investigated [7, 8]. Further in vitro studies using human thoracic spine specimens, which included the rib cage structures, determined the effect of discectomy [9–11], rib head resection [9, 11], facetectomy [9–11], laminectomy [11], transversal [10] and median sternotomy [12], costosternal release [10, 12], sternal fracture [13], the anterior rib cage except of the costovertebral joints [13, 14], and the superior rib cage [12]. All these studies concluded that the single rib cage structures have a significant effect on thoracic spine stability.

Nonetheless, there are still many open questions regarding the influence of the rib cage on the stability of the thoracic spine. For example, the monosegmental range of motion of the thoracic spine including the entire rib cage has never been investigated, nor has the effect of the rib cage on the biomechanics of the entire thoracic spine. Therefore, the objectives of the present study were: (1) To determine the stabilizing effect of specific rib cage structures (intercostal muscles, sternum, and costovertebral joints) on the entire human thoracic spine in order to calibrate and validate finite element models of the thoracic spine and rib cage system and to quantify the effect of standard surgical interventions (median longitudinal sternotomy, costovertebral release) on thoracic spine stability. (2) To examine the monosegmental ranges of motion of the thoracic spine in polysegmental testing with and without the rib cage. (3) To investigate the effect of the rib cage on coupled motions of the thoracic spine. Based on the previously published experiments, it was hypothesized that each specific structure has a significant effect on the stability of the thoracic spine.

## Materials and methods

### Study design

Six fresh frozen human thoracic spine specimens (C7-L1) including all rib cage structures with an average age of 56 years (range 50–65) were dissected from entire torsos (one male and five female). During preparation, specimens were scanned for ligament damage, bone fractures, and osteophytes. Bony, cartilaginous, and ligamentous structures as well as the intercostal muscles were left intact. The specimens were stored at -20°C and thawed at 6°C for 10 h prior to preparation and testing, which were both performed within 20 h to avoid disintegration. The cranial end of C7 and the caudal end of L1 were each embedded half in PMMA (Technovit 3040, Heraeus Kulzer, Werheim, Germany) for biomechanical testing. Prior to potting, screws were placed each in the cranial (C7) and caudal (L1) endplates to obtain a better fixation. Screws were also driven through the intervertebral disc between C7 and T1 to restrict the high mobility of this motion segment.

**Fig 1:**
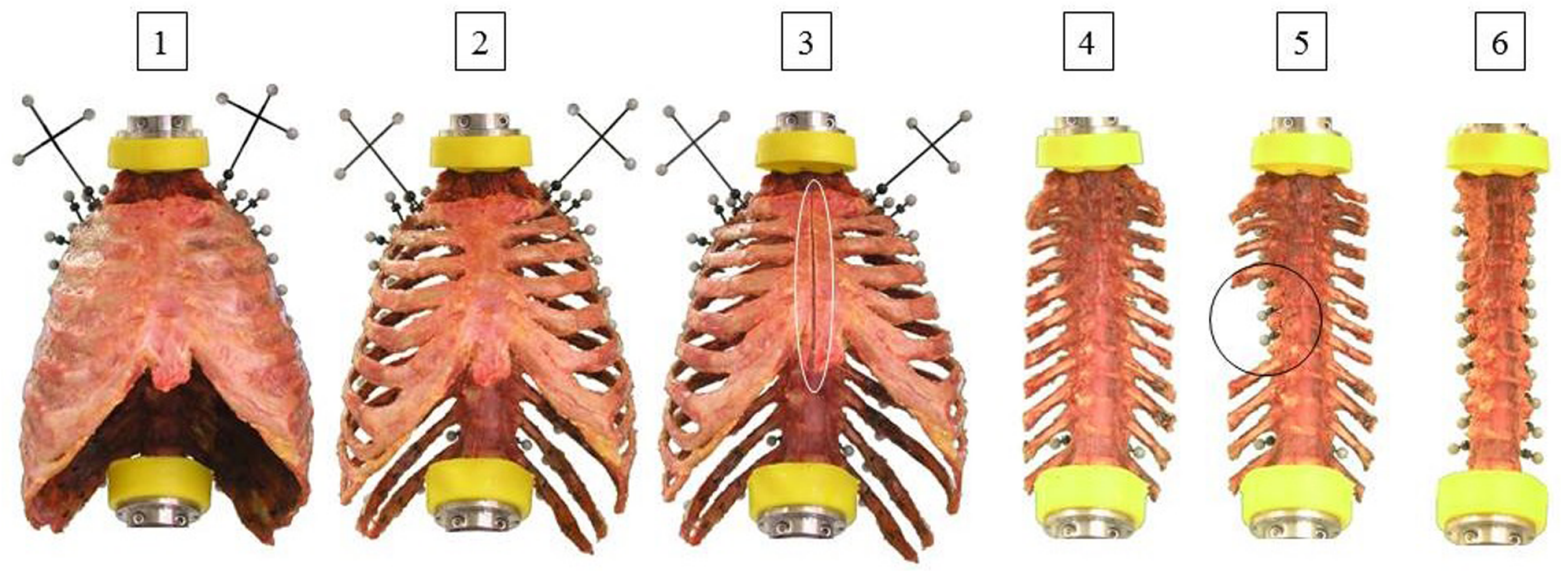
The six specimen conditions for biomechanical testing and motion analysis. Specimens were tested in intact condition (1), after removing the intercostal muscles (2), after median sternotomy (3), after removing the anterior rib cage up to rib stumps (4), after removing right sixth, seventh, and eighth rib head (5), as well as after removing all rib heads (6).

The test procedure was designed as a stepwise reduction of the single specimen structures whereby biomechanical testing and motion analysis between each reduction step was performed (Fig 1). The intercostal muscles and the rib heads were removed using a scalpel. Sixth to eighth rib head removal simulated the concept of costovertebral release, which provides good results in the surgical treatment of scoliosis [15]. Median longitudinal sternotomy and the removal of the anterior part of the rib cage up to the rib stumps were performed using an oscillating saw (OR-SY-518.01, Synthes®, Zuchwil, Switzerland). Median longitudinal sternotomy is a standard clinical intervention, which is performed in most cardiac surgeries such as cardiopulmonary bypass surgery or heart transplantation [16]. The destabilizing effect of median sternotomy on the thoracic spine has already been shown in a previous study, where the stabilizing effect of sternal closure was investigated [17].

The conduction of the study and the use of human specimens were approved by the ethical committee board of the University of Ulm, Germany (No. 302/14). The specimens were provided by the Anatomy Gifts Registry program (AGR, Hanover, Maryland, USA).

### Biomechanical testing

**Fig 2:**
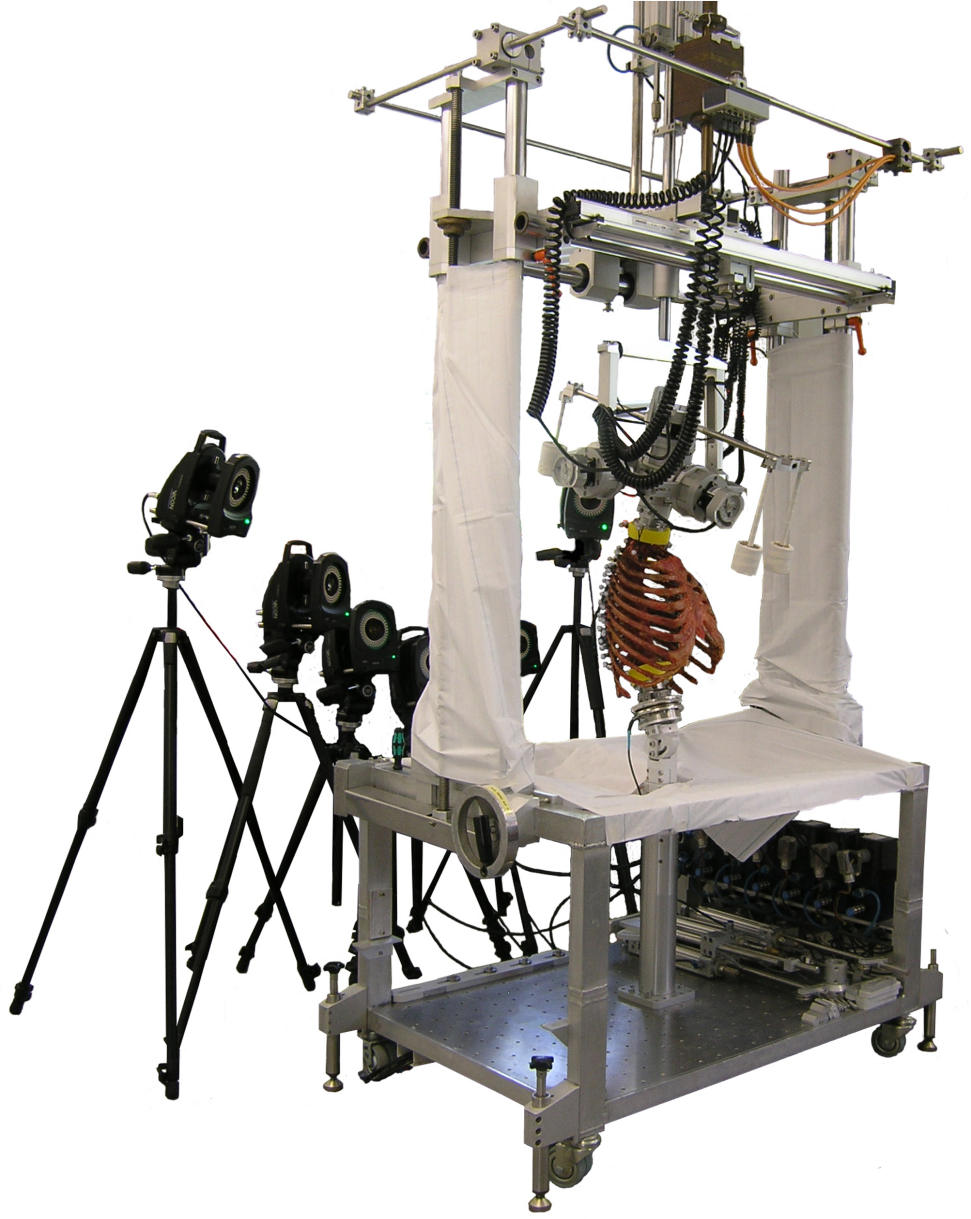
The experimental test setup. Pure moments of 2 Nm were applied in flexion/extension, lateral bending, and axial rotation using a custom-built spine tester [18]. Motion analysis was performed using an optical motion tracking system with six cameras.

The specimens were loaded quasi-statically on the cranial end (C7) with pure bending moments of ± 2 Nm in the three loading planes flexion/extension, lateral bending, and axial rotation (Fig 2) using a custom-built spine tester [18]. The loads were applied with a constant loading rate of 1.0°/s in flexion/extension and lateral bending as well as 0.5°/s in axial rotation to avoid viscoelastic superposition of the motion response [19]. To reduce viscoelastic effects, 3.5 loading cycles were performed in each loading plane (S1 Video), of which the last loading cycle was chosen for data evaluation.

The caudal end of the specimens (L1) was each fixed in an angle flange. Because the angle between the thoracic and lumbar spine is about 20° [20], the angle flange was positioned at 20° for four of the specimens with regular kyphosis and at 30° for two of the specimens with hyperkyphosis to reach a balanced, centered position within the spine tester.

### Motion analysis

**Fig 3:**
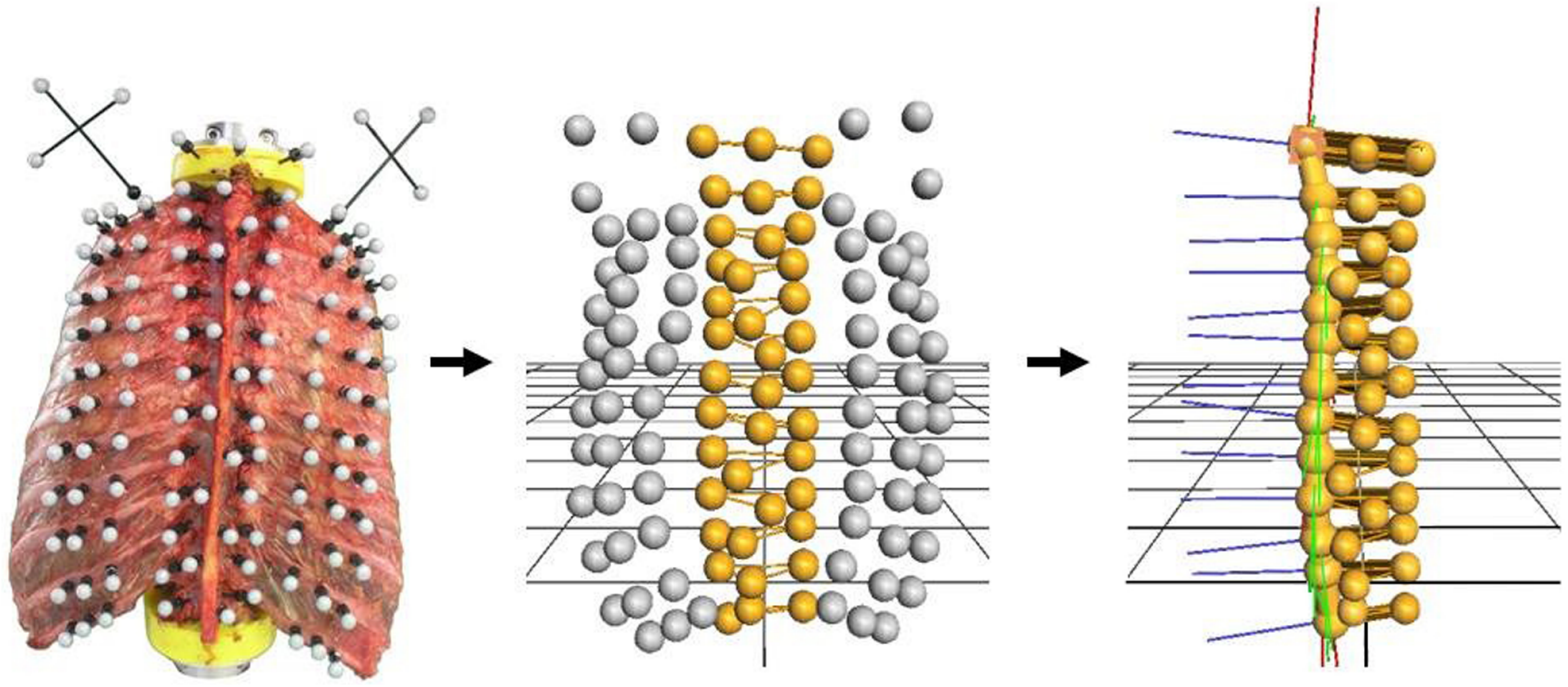
The process of motion analysis. Optical markers were transferred into a point cloud. Relative motions were determined by manual labeling.

Simultaneously with mechanical loading, motion data was captured using the optical motion tracking system Vicon MX13 (Vicon Motion Systems Ltd., Oxford, UK) consisting of six cameras. Motion analysis was performed to calculate range of motion (ROM) and neutral zone (NZ) together with the applied moment for the single functional spinal units (FSUs) from T1 to T12, as well as the coupled motions of the FSUs. All single vertebrae were therefore each equipped with three optical markers (Fig 3). The motion analysis used in the test setup was determined to have an accuracy of 0.1 mm or 0.1°, based on preliminary tests. Motion data was evaluated using Nexus 1.8.5 (Vicon Motion Systems Ltd., Oxford, UK, S2 Video) and processed with MATLAB R2014b (MathWorks, Natick, MA, USA) and Microsoft Excel 2010 (Microsoft Corp., Redmond, WA, USA).

ROM was defined as the maximum deflection of the single motion segments in one direction. NZ was defined as the intersection point of the hysteresis curve with the displacement axis at a bending moment of 0 Nm [21]. NZ represents the laxity of a specimen and specifies the region in which no or only small moments are generated during an applied displacement [21].

### Statistical analysis

Statistical tests were performed using the statistical analysis software SPSS 21 (IBM Corp., Armonk, NY, USA). ROM and NZ data were checked for normal distribution within the six specimen groups using Shapiro-Wilk normality test (p > 0.1) and then tested for significance (p < 0.05) using parametric Student’s t-test to evaluate differences between the six testing conditions. Because all ROM and NZ data were normally distributed, data were represented as mean with standard deviation. Holm-Bonferroni method was performed in order to account for the familywise error rate caused by multiple comparisons between the different groups. Finally, Cohen’s sample effect size (0.2 < d < 0.5: small effect, 0.5 < d < 0.8: mean effect, d > 0.8: strong effect) and observed power (1-β > 0.8) were evaluated to estimate the achieved statistical power of the results. Power analysis was performed post hoc using the Software G*Power [22]. Monosegmental ROM data were analyzed qualitatively and represented as median value to minimize the effect of outliers resulting from polysegmental testing. Coupled motions of the entire thoracic spine were also checked for normal distribution and represented as mean with standard deviation.

## Results

**Fig 4:**
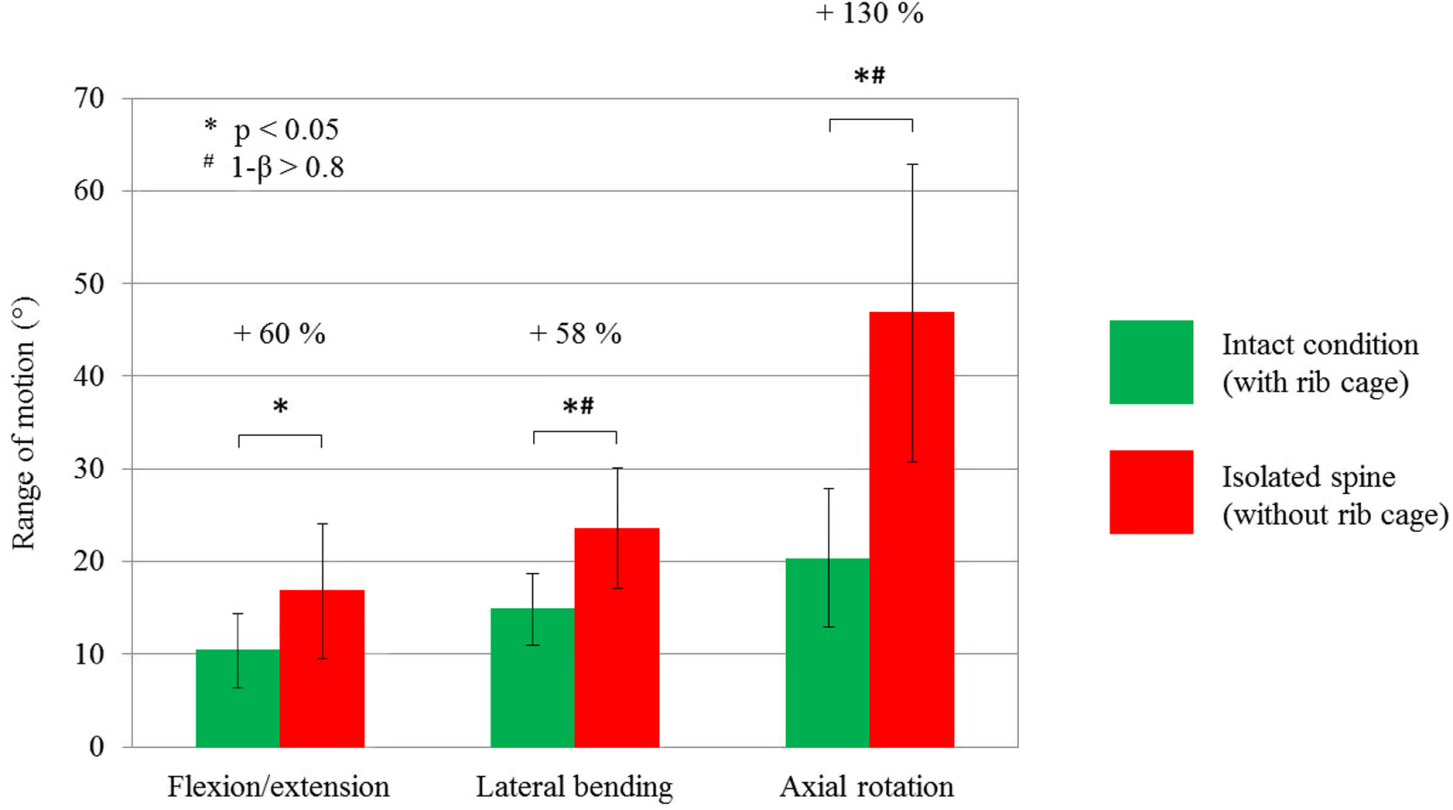
The ROM of the thoracic spine (T1-12, n = 6) for 2 Nm pure bending moment. The ROM is depicted as mean with standard deviation for each the intact condition with entire rib cage and the condition without entire rib cage in all loading directions.

The rib cage and its single structures were found to have a strong effect on thoracic spine rigidity (Table 1). Dissecting the intercostal muscles caused a significant increase of ROM and NZ in lateral bending (+ 22.4%, + 30.0%) and axial rotation (+ 22.6%, + 21.7%). Removing the anterior part of the rib cage up to rib stumps had the strongest effect on thoracic spine rigidity. Compared to the previous condition, ROM and NZ increased significantly in flexion/extension (+ 21.1%, + 25.3%), lateral bending (+ 9.8%, + 10.9%), and axial rotation (+ 30.1%, + 72.5%) without the anterior rib cage. Compared to the intact condition, the removal of the anterior part of the rib cage produced a significant increase in the ROM and NZ during flexion/extension (+ 52.2%, + 45.6%), lateral bending (+ 42.0%, + 54.0%), and axial rotation (+ 94.4%, + 187.8%), with a strong effect size (d > 0.8) in each loading direction, as well as a high power (1-β > 0.8) in axial rotation. Removing the entire rib cage increased the ROM significantly in all loading directions relative to the intact condition (Fig 4). ROM and NZ generally increased after each resection step except for the last resection step in flexion/extension.

**Table 1:** Ranges of motion (ROM) and neutral zones (NZ) of the thoracic spine (T1-T12) are shown for the single specimen conditions (IC = intact condition, W/o IM = without intercostal muscles, MST = after median sternotomy, W/o ARC = without anterior rib cage, W/o RH 6–8 = without 6th-8th right rib head, W/o RH = without all rib heads) in the single motion planes for 2 Nm pure bending moment (n = 6).

Specimen condition		Flexion/extension			Lateral bending			Axial rotation		
		M ± SD (°)	PC (%)	IC (%)	M ± SD (°)	PC (%)	IC (%)	M ± SD (°)	PC (%)	IC (%)
IC	ROM	10.5 ± 4.0			14.9 ± 3.9			20.4 ± 7.4		
	NZ	7.9 ± 3.4			11.8 ± 4.3			2.4 ± 1.1		
W/o IM	ROM	11.8 ± 5.0	+ 11.7 ^a^		18.3 ± 5.4	+ 22.4 *^b^		25.0 ± 9.5	+ 22.6 *^b^	
	NZ	8.9 ± 4.0	+ 12.6 *^a^		15.3 ± 5.6	+ 30.0 *^b^		3.0 ± 1.3	+ 21.7 *^a^	
MST	ROM	13.2 ± 5.6	+ 11.9 *^a^	+ 25.0 *^b^	19.3 ± 5.2	+ 5.7 ^a^	+ 29.3 *^c^	30.5 ± 11.1	+ 21.9 *^b^	+ 49.4 *^c^
	NZ	9.2 ± 4.1	+ 3.2	+ 16.2 *^a^	16.3 ± 5.9	+ 6.8 ^a^	+ 38.8 *^c^	4.1 ± 2.0	+ 37.1 ^b^	+ 66.9 *^c^
W/o ARC	ROM	16.0 ± 6.6	+ 21.1 *^b^	+ 52.2 *^c^	21.2 ± 5.7	+ 9.8 *^a^	+ 42.0 *^c^	39.7 ± 13.4	+ 30.1 *^c^	+ 94.4 *^c^^#^
	NZ	11.5 ± 5.4	+ 25.3 *^b^	+ 45.6 *^c^	18.1 ± 6.1	+ 10.9 *^a^	+ 54.0 *^c^	7.0 ± 3.3	+ 72.5 *^c^	+ 187.8 *^c^^#^
W/o RH 6–8	ROM	17.0 ± 7.5	+ 6.3	+ 6.3	22.3 ± 5.7	+ 5.3 ^a^	+ 49.6 *^c^^#^	44.3 ± 15.2	+ 11.7 *^a^	+ 117.1 *^c^^#^
	NZ	12.5 ± 6.4	+ 8.9	+ 8.9	19.4 ± 6.1	+ 7.3 ^a^	+ 65.2 *^c^^#^	8.3 ± 3.9	+ 18.5 ^a^	+ 240.9 *^c^^#^
W/o RH	ROM	16.9 ± 7.3	- 0.6	- 0.6	23.6 ± 6.4	+ 5.7 ^a^	+ 58.0 *^c^^#^	46.9 ± 16.1	+ 5.8 *	+ 129.7 *^c^^#^
	NZ	11.7 ± 5.9	- 6.3	- 6.3	20.6 ± 7.0	+ 6.3 ^a^	+ 75.5 *^c^^#^	8.6 ± 4.6	+ 4.1 ^a^	+ 254.9 *^c^^#^

M = mean, SD = standard deviation, PC = variation compared to previous condition, IC = variation compared to intact condition

* p < 0.05

^a^ small effect (0.2 < d < 0.5

^b^ mean effect (0.5 < d < 0.8)

^c^ strong effect (d > 0.8)

^#^ high power (1-β > 0.8)

Performing a median sternotomy increased the ROM significantly in flexion/extension and axial rotation compared to its previous condition without intercostal muscles, as previously shown [17]. Compared to the intact condition, ROM and NZ were significantly increased after a median sternotomy in flexion/extension (+ 25.0%, + 16.2%), lateral bending (+ 29.3%, + 38.8%), and axial rotation (+ 49.4%, + 66.9%). Removing the sixth to eighth right rib head increased the ROM significantly in axial rotation (+ 11.7%) relative to its previous condition with all costovertebral joints left intact.

Removing the entire rib cage generally increased the monosegmental ROM, except for the lower two segments T10-T11 (in lateral bending) and T11-T12 (in all three directions) (Table 2). In flexion/extension and lateral bending, the highest flexibility was detected in T1-T2, decreasing in the inferior direction. In axial rotation, the flexibility of the FSUs was more evenly distributed over the entire thoracic spine, but increased much more in the superior half after removing the entire rib cage compared to its intact condition. Because of inverse motions of the functional spinal units, the monosegmental ROM were found to be negative in some cases, especially in flexion/extension and generally in the condition without the entire rib cage.

**Table 2:** Ranges of motion are shown for intact condition (= IC) and the condition without the entire rib cage (= W/o RC) in the single motion planes for all functional spinal units and a pure bending moment of 2 Nm (n = 6).

	Flexion/extension		Lateral bending		Axial rotation	
	IC	W/o RC	IC	W/o RC	IC	W/o RC
	Mdn (°)	Mdn (°)	Mdn (°)	Mdn (°)	Mdn (°)	Mdn (°)
	Max / Min (°)	Max / Min (°)	Max / Min (°)	Max / Min (°)	Max / Min (°)	Max / Min (°)
T1-T2	3.4	4.7	2.8	4.8	2.9	6.2
	4.4 /2.7	6.4 / 3.1	4.7 / 2.2	7.0 / 3.4	3.7 / 1.4	8.0 / 3.8
T2-T3	1.3	2.4	2.4	3.5	1.7	6.4
	2.6 / -1.3	3.2 / -0.2	2.8 / 0.6	5.2 / 0.7	3.3 / 0.5	7.3 / 0.2
T3-T4	1.3	1.9	2.1	3.5	1.0	4.5
	1.9 / 0.4	3.7 / 1.0	3.4 / 1.2	5.2 / 2.1	1.1 / 0.3	7.8 / 2.4
T4-T5	0.5	1.8	1.8	3.8	1.3	5.0
	1.1 / -0.8	4.1 / -0.4	2.9 / 0.6	4.5 / 1.3	1.8 / 0.3	9.6 / 1.9
T5-T6	0.3	0.7	1.3	2.9	0.8	5.1
	0.9 / 0.2	2.0 / 0.5	2.0 / 0.6	3.9 / 1.2	1.8 / 0.4	7.7 / 1.9
T6-T7	0.6	1.8	1.1	2.0	2.2	5.8
	2.8 / -0.5	3.7 / -0.5	1.7 / 0.5	3.6 / 0.6	2.7 / 0.5	6.9 / 0.8
T7-T8	0.1	1.0	0.8	1.4	1.7	2.9
	1.0 / -0.8	1.6 / -0.2	1.2 / 0.5	3.0 / 1.0	2.2 / 0.8	6.3 / -0.6
T8-T9	1.0	1.1	0.9	1.4	3.0	5.9
	1.8 / -1.1	2.4 / -2.6	1.4 / 0.6	2.1 / 0.5	4.2 / 0.7	6.9 / 1.3
T9-T10	0.9	1.0	0.8	1.1	3.8	4.7
	2.0 / 0.0	1.8 / -0.7	1.6 / 0.4	1.7 / 0.1	5.3 / 1.0	6.3 / 1.7
T10-T11	0.8	1.0	0.6	0.2	2.6	2.8
	2.7 / 0.2	4.1 / 0.2	1.1 / 0.0	0.6 / -0.1	4.3 / 0.5	5.7 / 0.4
T11-T12	0.7	0.1	0.4	0.0	0.9	0.8
	1.1 / -0.3	1.9 / -4.7	0.7 / 0.2	0.3 / -0.5	2.5 / 0.6	2.6 / 0.6

Mdn = median, Max = maximum, Min = minimum

Coupled motions of the thoracic spine with the intact rib cage were detected mainly in lateral bending, with a large coupled axial rotation in the opposite direction, as well as in axial rotation with a slight coupled lateral bending in the same direction (Table 3). After removal of the entire rib cage, these coupled motions increased approximately proportionally to the increasing ROM of the main loading directions.

**Table 3:** Coupled motion ROM of the thoracic spine (T1-T12, n = 6) are shown for intact condition (= IC) and condition without the entire rib cage (= W/o RC) in the six main loading directions.

Specimen condition		Flexion	Extension	Lat. bend. left	Lat. bend. right	Ax. rot. left	Ax. rot. right
		M ± SD (°)	M ± SD (°)	M ± SD (°)	M ± SD (°)	M ± SD (°)	M ± SD (°)
IC	Coupled flexion	5.2 ± 1.7		0.2 ± 0.5			
	Coupled extension		5.3 ± 2.4		0.3 ± 0.5	0.1 ± 0.8	0.1 ± 0.7
	Coupled lat. bend. left		0.1 ± 0.3	7.5 ± 1.9		1.1 ± 1.2	
	Coupled lat. bend. right	0.1 ± 0.3			7.5 ± 1.9		1.1 ± 1.3
	Coupled ax. rot. left		0.2 ± 0.4		4.6 ± 2.3	10.2 ± 3.7	
	Coupled ax. rot. right	0.2 ± 0.4		4.6 ± 2.3			10.2 ± 3.7
W/o RC	Coupled flexion	8.5 ± 4.0		0.3 ± 1.2	0.4 ± 1.7		
	Coupled extension		8.4 ± 3.4			0.1 ± 1.2	1.6 ± 1.2
	Coupled lat. bend. left	0.2 ± 0.5		11.8 ± 3.2		2.2 ± 2.4	
	Coupled lat. bend. right		0.2 ± 0.5		11.8 ± 3.2		2.2 ± 2.4
	Coupled ax. rot. left		1.2 ± 1.5		6.4 ± 3.4	23.5 ± 8.1	
	Coupled ax. rot. right	1.6 ± 1.5		6.3 ± 3.3			23.5 ± 8.1

M = mean, SD = standard deviation

## Discussion

Detailed knowledge of the biomechanics of the thoracic spine and the effect of the rib cage on its stability is essential for the improvement of surgical interventions, as well as therapeutic treatments in the thoracic region. Numerical models of the thoracic spine including the rib cage may help to understand and optimize the effects of specific medical procedures, but need to be validated by experimental data [1, 2]. Several in vitro studies investigated the effect of specific rib cage structures on thoracic spine stability using multiple kinds of test setups and few resection steps [7–14]. The purpose of the present study was therefore to investigate the stabilizing effect of the rib cage on the thoracic spine and to provide data in order to calibrate and validate finite element models of the thoracic spine including the rib cage.

The results of the present study showed that all rib cage structures contribute to thoracic spine stability. The strong stabilizing effect of the rib cage becomes distinct especially when looking at the difference between the intact rib cage and the isolated spine (Fig 4), which has never been investigated before. These findings suggest that the rib cage is of prime importance regarding the biomechanical integrity of the spine, since the thoracic spine is additionally stabilized by the ribs and the costovertebral ligaments, in contrast to the cervical and lumbar spine, which are mainly stabilized by the adjacent musculature. Therefore, it can be assumed that the rib cage, in addition to its function as protector for the inner organs, respiration support, and framework for the insertion of muscles, has the role of a flexibility limiter for the spine in order to prevent damage caused by high bending moments during movements.

The stabilizing effect of the intercostal muscles, also never previously investigated in vitro, was especially evident during lateral bending and axial rotation, when these muscles were stressed in tension. Intercostal muscles should therefore be left intact for biomechanical testing and should be included in computational models of the rib cage. Removing the anterior part of the rib cage up to the rib stumps had the strongest effect on thoracic spine stability of all resection steps performed in the present study, as well as any former in vitro studies (Table 4). Watkins et al. detected the strongest effect in flexion/extension using a biaxial testing device without applying pure moments [13], whereas Mannen et al. observed the strongest effect in axial rotation [14], which corresponds to the results of the present study. The sternal complex, which connects most of the single ribs with each other anteriorly, seems therefore to be of high importance for the rigidity of the thoracic spine regarding the effect of the rib cage.

**Table 4:** Comparison of ROM increase (%) after removing the anterior rib cage up to rib stumps relative to intact condition with literature data.

	Flexion/extension	Lateral bending	Axial rotation
Present study	+ 52.2	+ 42.0	+ 94.4
Watkins et al. [13]	+ 66.1	+ 54.8	+ 45.9
Mannen et al. [14]	+ 18.6	+ 37.4	+ 77.0

The destabilizing effect of median sternotomy on the thoracic spine, especially in axial rotation, was also detected in the study of Brasiliense et al. [12], although relatively smaller compared to the present study. This might be due to their test setup, in which only the upper four segments were tested, while relative motions between the ribs and vertebrae were restricted. Combining the findings of these two studies, sternal closure is recommended after surgical transection of the sternum from a biomechanical perspective to prevent spinal destabilization. The removal of the right sixth to eighth rib head only produced a significant effect in axial rotation compared to its previous condition with all costovertebral joints left intact. This agrees with the study of Feiertag et al., who also did not find a significant increase in the ROM after removal of the right ninth rib head under flexion/extension as well as lateral bending [9]. In contrast, Oda et al. detected a significant increase in the ROM after right rib head removal in all loading planes using thoracic functional spinal units [11]. However, since Oda et al. performed a radical discectomy before rib head removal, the results are not directly comparable. Nevertheless, partial costovertebral release seems to destabilize the thoracic spine at least in axial rotation. This might facilitate the surgical correction of scoliosis, whereby a spinal fixation is recommended to avoid spinal instability.

Compared to the literature, monosegmental ROM of the present study was quite low in flexion/extension and lateral bending (Table 5). This might indicate that higher bending moments than 2 Nm and monosegmental testing provide a more physiologic simulation of the in vivo situation in these two loading directions, especially in the lower segments where the intervertebral discs exhibit larger cross sections. Also the high NZ to ROM ratios in flexion/extension and lateral bending (Table 1) suggest that the flexibility of the thoracic spine is much higher in vivo. The results of the present study also indicate that the lower two pairs of ribs do not have a significant effect on thoracic spine stability, since the costovertebral joints of the lower two pairs of ribs are not located at the level of the intervertebral disc and therefore do not provide intersegmental stability.

**Table 5:** Comparison of monosegmental ROM with literature data.

	Flexion/Extension						Lateral bending					Axial rotation					
	IC	CVJ	[23]	[24]	[25]	[26]	IC	CVJ	[23]	[24]	[25]	IC	CVJ	[23]	[24]	[25]	[27]
T1-T2	3.4	4.7	4.0	2.8	5.6	3.9	2.8	4.8	10.0	6.0	4.0	2.9	6.2	18.0	-	9.1	2.4
T2-T3	1.3	2.4	4.0	2.6	5.6	2.6	2.4	3.5	12.0	4.8	4.0	1.7	6.4	16.0	4.0	9.1	3.2
T3-T4	1.3	1.9	4.0	2.3	5.6	1.2	2.1	3.5	10.0	3.7	4.0	1.0	4.5	16.0	5.1	9.1	2.8
T4-T5	0.5	1.8	4.0	1.8	4.9	0.9	1.8	3.8	12.0	5.0	4.1	1.3	5.0	16.0	3.9	11.7	3.2
T5-T6	0.3	0.7	4.0	2.6	4.9	1.5	1.3	2.9	12.0	5.3	4.1	0.8	5.1	16.0	5.0	11.7	3.6
T6-T7	0.6	1.8	5.0	2.3	4.9	2.1	1.1	2.0	12.0	5.9	4.1	2.2	5.8	14.0	4.1	11.7	3.8
T7-T8	0.1	1.0	6.0	3.3	4.9	1.9	0.8	1.4	12.0	4.1	4.1	1.7	2.9	14.0	4.3	11.7	4.6
T8-T9	1.0	1.1	6.0	3.2	5.4	2.7	0.9	1.4	12.0	4.7	6.2	3.0	5.9	12.0	5.5	4.6	5.0
T9-T10	0.9	1.0	6.0	3.1	5.4	3.3	0.8	1.1	12.0	4.4	6.2	3.8	4.7	8.0	3.2	4.6	5.4
T10-T11	0.8	1.0	9.0	3.9	5.4	3.6	0.6	0.2	14.0	4.4	6.2	2.6	2.8	4.0	3.4	4.6	5.2
T11-T12	0.7	0.1	12.0	6.5	5.4	3.8	0.4	0.0	18.0	3.7	6.2	0.9	0.8	4.0	2.6	4.6	2.6
Total	10.5	16.9	64.0	34.4	57.8	27.5	14.9	23.6	136.0	52.0	53.0	20.4	46.9	138.0	41.1	92.0	41.8

IC = Intact condition (present study), CVJ = Condition without rib cage and costovertebral joints (present study), [23] = White and Panjabi (1990, literature collection), [24] = White et al. (1969, in vitro, polysegmental, without ribs, maximum ROM), [25] = Willems et al. (1996, in vivo, maximum ROM), [26] = Morita et al. (2014, in vivo, maximum ROM), [27] = Fujimori et al. (2012, in vivo, maximum ROM)

In the present study, lateral bending in one direction induced a considerable axial rotation in the opposite direction. This was also observed in the study of Brasiliense et al. in the upper third of the thoracic spine [12], whereas Willems et al. determined coupled axial rotation in same direction [25]. The slight coupled lateral bending in the same direction during axial rotation observed in the present study is in accordance with the results of former in vivo studies [25, 27]. Coupled motions of the thoracic spine can be caused by several factors, for instance by the specific orientation of the facet joints, the morphology of the intervertebral discs, ligamentous structures, or the ribs. The different coupled motion behavior during lateral bending and axial rotation in our study is probably due to morphological characteristics of the thoracic spine and rib cage. This complex motion behavior should be analyzed separately by means of monosegmental testing of the thoracic spine including the ribs. Because of the alternating motion pattern, monosegmental coupled motions were not quantified in the present study, but it is known that coupled motions of thoracic spinal segments decrease in the inferior direction [23, 27].

The present study entailed some limitations. Statistical differences and observed power of the results were quite low, which can be traced to the small specimen number (n = 6). However, increasing the number of specimens to achieve higher power is also related with ethical and financial issues. Moreover, the present study primarily intended to provide data for numerical modeling. Statistical significance could have been also increased without using the Holm-Bonferroni method, which is critically discussed in research [28]. The relatively high standard deviations of the results followed from the interindividual differences of the single specimens, which showed each a similar motion behavior in relative terms. Another limitation of the present study was the polysegmental testing, which is just qualitatively comparable to monosegmental testing, since the ROM is tending to be smaller in polysegmental testing [29]. Monosegmental ROM was also sometimes found to show an inverse motion behavior in the present study and should therefore be tested separately in a monosegmental test setup with associated rib and sternal portion. Including a follower load in the test setup, which is sometimes stipulated in thoracic spine research [30], may improve the monosegmental motion behavior, but it would also increase the specimen stiffness and the number of boundary conditions in a numerical model validation. In the present study, pure bending moments of 2 Nm were applied, which correspond to former in vitro studies using a polysegmental test setup for the thoracic spine [11, 13], whereas generally pure moments of 5 Nm are recommended for the thoracic region in spine research [21]. Because of specimen length and limited testing device dimensions, loading of the specimens with 5 Nm was not feasible in flexion/extension and lateral bending. However, it was imperative to perform polysegmental testing when evaluating the effect of the entire rib cage on thoracic spine stability. Due to its in vitro nature, the results of the present study are not directly comparable to studies which were performed in vivo, although it was previously shown that the application of pure moments can simulate physiological loading conditions [31]. In future studies, muscle forces should therefore be included in the experimental setup to simulate an even more physiological motion behavior of the thoracic spine and the rib cage.

## Conclusions

The rib cage and its single structures have a significant stabilizing effect on the thoracic spine. Therefore, the rib cage should be regarded in clinical scenarios, in vitro, as well as in numerical models of the thoracic spine. Using the biomechanical data of the present study, numerical models of the thoracic spine including the rib cage can be calibrated and validated.

## Supporting information

S1 Dataset Raw data of the in vitro experiments and statistical analysis. Raw data of the experiments (polysegmental Rom/NZ, monosegmental ROM, and coupled motions ROM) and statistical analysis (t-test, Holm-Bonferroni procedure, effect size test, and power analysis) are shown.
(XLSX)

S1 Video Load application. The application of ±2 Nm pure moment is shown in time lapse exemplarily for axial rotation in the intact condition.
(MP4)

S2 Video Optical marker motion. The virtual motion of the optical markers during load application is shown.
(MP4)

## References

[1] Andriacchi T, Schultz A, Belytschko T, Galante J. A model for studies of mechanical interactions between the human spine and rib cage. J Biomech. 1974;7(6):497–507. 4452675 10.1016/0021-9290(74)90084-0

[2] Sham ML, Zander T, Rohlmann A, Bergmann G. Effects of the rib cage on thoracic spine flexibility. Biomed Tech (Berl). 2005;50(11):361–5. 16370149 10.1515/BMT.2005.051

[3] Adams MA, Hutton WC, Stott JRR. The resistance to flexion of the lumbar intervertebral joint. Spine (Phila Pa 1976). 1980;5(3):245–53. 7394664 10.1097/00007632-198005000-00007

[4] Heuer F, Schmidt H, Klezl Z, Claes L, Wilke HJ. Stepwise reduction of functional spinal structures increase range of motion and change lordosis angle. J Biomech. 2007;40(2):271–80. doi: 10.1016/j.jbiomech.2006.01.007 16524582 10.1016/j.jbiomech.2006.01.007

[5] Panjabi MM, White A III, Johnson RM. Cervical spine mechanics as a function of transection of components. J Biomech. 1975;8(5):327–36. 1184604 10.1016/0021-9290(75)90085-8

[6] Posner I, White AA III, Edwards WT, Hayes WC. A biomechanical analysis of the clinical stability of the lumbar and lumbosacral spine. Spine (Phila Pa 1976). 1982;7(4):374–89. 7135070 10.1097/00007632-198207000-00008

[7] Oda I, Abumi K, Lü D, Shono Y, Kaneda K. Biomechanical role of the posterior elements, costovertebral joints, and rib cage in the stability of the thoracic spine. Spine (Phila Pa 1976). 1996;21(12):1423–9. 8792518 10.1097/00007632-199606150-00005

[8] Takeuchi T, Abumi K, Shono Y, Oda I, Kaneda K. Biomechanical role of the intervertebral disc and costovertebral joint in stability of the thoracic spine: a canine model study. Spine (Phila Pa 1976). 1999;24(14):1414–20. 10423785 10.1097/00007632-199907150-00005

[9] Feiertag MA, Horton WC, Norman JT, Proctor FC, Hutton WC. The effect of different surgical releases on thoracic spinal motion: a cadaveric study. Spine (Phila Pa 1976). 1995;20(14):1604–11. 7570176 10.1097/00007632-199507150-00009

[10] Horton WC, Kraiwattanapong C, Akamaru T, Minamide A, Park JS, Park MS, et al The role of the sternum, costosternal articulations, intervertebral disc, and facets in thoracic sagittal plane biomechanics: a comparison of three different sequences of surgical release. Spine (Phila Pa 1976). 2005;30(18):2014–23. 16166888 10.1097/01.brs.0000180478.96494.88

[11] Oda I, Abumi K, Cunningham BW, Kaneda K, McAfee PC. An in vitro human cadaveric study investigating the biomechanical properties of the thoracic spine. Spine (Phila Pa 1976). 2002;27(3):E64–70. 11805710 10.1097/00007632-200202010-00007

[12] Brasiliense LB, Lazaro BC, Reyes PM, Dogan S, Theodore N, Crawford NR. Biomechanical contribution of the rib cage to thoracic stability. Spine (Phila Pa 1976). 2011;36(26):E1686–93. 22138782 10.1097/BRS.0b013e318219ce84

[13] Watkins R IV, Watkins R III, Williams L, Ahlbrand S, Garcia R, Karamanian A, et al Stability provided by the sternum and rib cage in the thoracic spine. Spine (Phila Pa 1976). 2005;30(11):1283–6. 15928553 10.1097/01.brs.0000164257.69354.bb

[14] Mannen EM, Anderson JT, Arnold PM, Friis EA. Mechanical contribution of the rib cage in the human cadaveric thoracic spine. Spine (Phila Pa 1976). 2015;40(13):E760–6. 25768687 10.1097/BRS.0000000000000879

[15] Li C, Fu Q, Zhou Y, Yu H, Zhao G. Surgical treatment of severe congenital scoliosis with unilateral unsegmented bar by concave costovertebral joint release and both-ends wedge osteotomy via posterior approach. Eur Spine J. 2012;21(3):498–505. doi: 10.1007/s00586-011-1972-6 21863460 10.1007/s00586-011-1972-6 PMC3296859

[16] Dalton ML, Connally SR, Sealy WC. Julian's reintroduction of Milton's operation. Ann Thorac Surg. 1992;53(3):532–3. 1540081 10.1016/0003-4975(92)90293-d

[17] Liebsch C, Graf N, Wilke HJ. Eur Spine J. 2016;

[18] Wilke HJ, Claes L, Schmitt H, Wolf S. A universal spine tester for in vitro experiments with muscle force simulation. Eur Spine J. 1994;3(2):91–7. 7874556 10.1007/BF02221446

[19] Wilke HJ, Jungkunz B, Wenger K, Claes LE. Spinal segment range of motion as a function of in vitro test conditions: effects of exposure period, accumulated cycles, angular‐deformation rate, and moisture condition. Anat Rec. 1998;251(1):15–9. 9605215 10.1002/(SICI)1097-0185(199805)251:1<15::AID-AR4>3.0.CO;2-D

[20] Roussouly P, Gollogly S, Berthonnaud E, Dimnet J. Classification of the normal variation in the sagittal alignment of the human lumbar spine and pelvis in the standing position. Spine (Phila Pa 1976). 2005;30(3):346–53. 15682018 10.1097/01.brs.0000152379.54463.65

[21] Wilke HJ, Wenger K, Claes L. Testing criteria for spinal implants: recommendations for the standardization of in vitro stability testing of spinal implants. Eur Spine J. 1998;7(2):148–54. doi: 10.1007/s005860050045 9629939 10.1007/s005860050045 PMC3611233

[22] Faul F, Erdfelder E, Buchner A, Lang AG. Statistical power analyses using G*Power 3.1: tests for correlation and regression analyses. Behav Res Methods. 2009;4:1149–60. 10.3758/BRM.41.4.1149 19897823

[23] White AA III, Panjabi MM. Clinical biomechanics of the spine. 2nd ed. Philadelphia, PA: Lippincott; 1990.

[24] White AA III. Analysis of the mechanics of the thoracic spine in man: an experimental study of autopsy specimens. Acta Orthop Scand. 1969;40(sup127):1–105. 10.3109/ort.1969.40.suppl-127.01 5264709

[25] Willems JM, Jull GA, Ng JF. An in vivo study of the primary and coupled rotations of the thoracic spine. Clin Biomech. 1996;11(6):311–6. 10.1016/0268-0033(96)00017-4 11415638

[26] Morita D, Yukawa Y, Nakashima H, Ito K, Yoshida G, Machino M, et al Range of motion of thoracic spine in sagittal plane. Eur Spine J. 2014;23(3):673–8. doi: 10.1007/s00586-013-3088-7 24217984 10.1007/s00586-013-3088-7 PMC3940794

[27] Fujimori T, Iwasaki M, Nagamoto Y, Ishii T, Kashii M, Murase T, et al Kinematics of the thoracic spine in trunk rotation: in vivo 3-dimensional analysis. Spine (Phila Pa 1976). 2012;37(21):E1318–28. 22772578 10.1097/BRS.0b013e318267254b

[28] Nakagawa S. A farewell to Bonferroni: the problems of low statistical power and publication bias. Behav Ecol. 2004;15(6):1044–5.

[29] Kettler A, Wilke HJ, Haid C, Claes L. Effects of specimen length on the monosegmental motion behavior of the lumbar spine. Spine (Phila Pa 1976). 2000;5(5):543–50. 10.1097/00007632-200003010-00003 10749629

[30] Sis HL, Mannen EM, Wong BM, Cadel ES, Bouxsein ML, Anderson DE, et al Effect of follower load on motion and stiffness of the human thoracic spine with intact rib cage. (2016). J Biomech. [Epub ahead of print]. 10.1016/j.jbiomech.2016.08.003 PMC5702885 27545081

[31] Wilke HJ, Rohlmann A, Neller S, Schultheiß M, Bergmann G, Graichen F, et al Is it possible to simulate physiologic loading conditions by applying pure moments? A comparison of in vivo and in vitro load components in an internal fixator. Spine (Phila Pa 1976). 2001;26(6):636–42. 11246374 10.1097/00007632-200103150-00014

